# Hsa-miR-326 targets *CCND1* and inhibits non-small cell lung cancer development

**DOI:** 10.18632/oncotarget.7071

**Published:** 2016-01-29

**Authors:** Chengcao Sun, Chuanfeng Huang, Shujun Li, Cuili Yang, Yongyong Xi, Liang Wang, Feng Zhang, Yunfeng Fu, Dejia Li

**Affiliations:** ^1^ Department of Occupational and Environmental Health, School of Public Health, Wuhan University, 430071 Wuhan, P.R.China; ^2^ Department of Pharmacology, Basic Medical School, Nanyang Medical College, 473003 Nanyang, P.R.China; ^3^ Wuhan Hospital for the Prevention and Treatment of Occupational Diseases, 430071 Wuhan, P.R.China; ^4^ The Third Xiang-Ya Hospital, Central South University, 410013 Changsha, P.R.China

**Keywords:** Hsa-miRNA-326 (miR-326), cyclin D1, non-small cell lung cancer (NSCLC), proliferation, apoptosis

## Abstract

Hsa-miRNA-326 (miR-326) has recently been discovered having anticancer efficacy in different organs. However, the role of miR-326 on non-small cell lung cancer (NSCLC) is still ambiguous. In this study, we investigated the role of miR-326 on the development of NSCLC. The results indicated that miR-326 was significantly down-regulated in primary tumor tissues and very low levels were found in NSCLC cell lines. Ectopic expression of miR-326 in NSCLC cell lines significantly suppressed cell growth as evidenced by cell viability assay, colony formation assay and BrdU staining, through inhibition of cyclin D1, cyclin D2, CDK4 and up-regulation of p57(Kip2) and p21(Waf1/Cip1). In addition, miR-326 induced apoptosis, as indicated by concomitantly with up-regulation of key apoptosis protein cleaved caspase-3, and down-regulation of anti-apoptosis protein Bcl2. Moreover, miR-326 inhibited cellular migration and invasiveness through inhibition of matrix metalloproteinases (MMP)-7 and MMP-9. Further, oncogene *CCND1* was revealed to be a putative target of miR-326, which was inversely correlated with miR-326 expression in NSCLC. Taken together, our results demonstrated that miR-326 played a pivotal role on NSCLC through inhibiting cell proliferation, migration, invasion, and promoting apoptosis by targeting oncogenic *CCND1*.

## INTRODUCTION

MicroRNAs (miRNAs) are a class of small, highly conserved, and non-coding RNAs that directly bind to some sequence-specific sites of target genes' 3′-UTRs (3′ untranslated regions), which lead to inhibition of these genes expression [1–3]. Increasing evidences have confirmed that ectopic miRNAs are key regulatory factors in various types of cancers [4–8]. Selective miRNA expression contributes to tumor proliferation, apoptosis, senescence, cell identity, stem cell maintenance and metastasis [7, 9–15]. Though recent researches of miRNAs have brought mind-blowing insight into our knowledge of human cancers, there are still large amount of unknown details that need to be explored further.

Lung cancer is one of the most frequently diagnosed cancers and is the leading cause of cancer-associated death both in men and women around the world. There are estimated to be 1.80 million new cases in 2012, killing about 1.59 million people per year globally, extrapolating from a 2012 International Agency for Research on Cancer (IARC) risk assessment [16], and this trend is expected to continue until 2030. Generally, approximately 85% of lung cancers are classified histopathologically as non-small cell lung carcinomas (NSCLC). Treatment advances have been made with the use of platinum-based chemotherapy [17], but the 5-year overall survival (OS) rate of just 16% for all stages [18]. These changes are attributed to silencing of tumor suppressor genes, dysregulation of proto-oncogenes, and an up-regulation of genes that promote cell growth and transformation and ultimately tumor development [19–23].

MiR-326, a recognized tumor-suppressing miRNA, has been shown to be down-regulated in a variety of diseases including cancers, such as pulmonary fibrosis [24], multiple sclerosis [25], colorectal cancer [26], breast cancer [27], glioma [28–30], glioblastoma [31] and brain tumors [32]. It is also reported that miR-326 associates with biochemical markers of bone turnover in lung cancer bone metastasis as well as promotes EMT-induced cells invasion in lung adenocarcinoma [33]. Wang *et al.* reported that miR-326 is expressed abnormally between the non-small cell lung cancer metastatic and non-metastatic tissues, which provides experimental basis for exploring the mechanism of non-small cell lung cancer metastasis and provides a potential idea for molecular diagnosis and treatment [34]. These results suggest tumor-suppressive functions of miR-326 in lung cancer but up to now this suggestion has not been rigorously tested.

The goal for our current study is to investigate the biological functions of miR-326 in lung cancer and to explore the underlying mechanisms of action. We show for the first time that miR-326 directly targets and regulates the full-length 3′-UTR of the human CCND1 mRNA, which is up-regulated in many cancers, including lung cancer. cyclin D1 is encoded by *CCND1* gene, and plays a key role in the control of invasive growth during tumorigenesis [35]. Here, we reported that miR-326 is indeed suppressed in primary lung cancers compared with the matching adjacent normal tissues, and found 3′-UTR of the human CCND1 mRNA is really a target of miR-326. Collectively, we discovered that miR-326 inhibits NSCLC cell growth, migration, invasion and colony formation, and promoted cell apoptosis by targeting 3′-UTR of *CCND1*.

## RESULTS

### MiR-326 is down-regulated in primary human lung cancer and NSCLC cell lines, and benefits for prognosis

**Figure 1:**
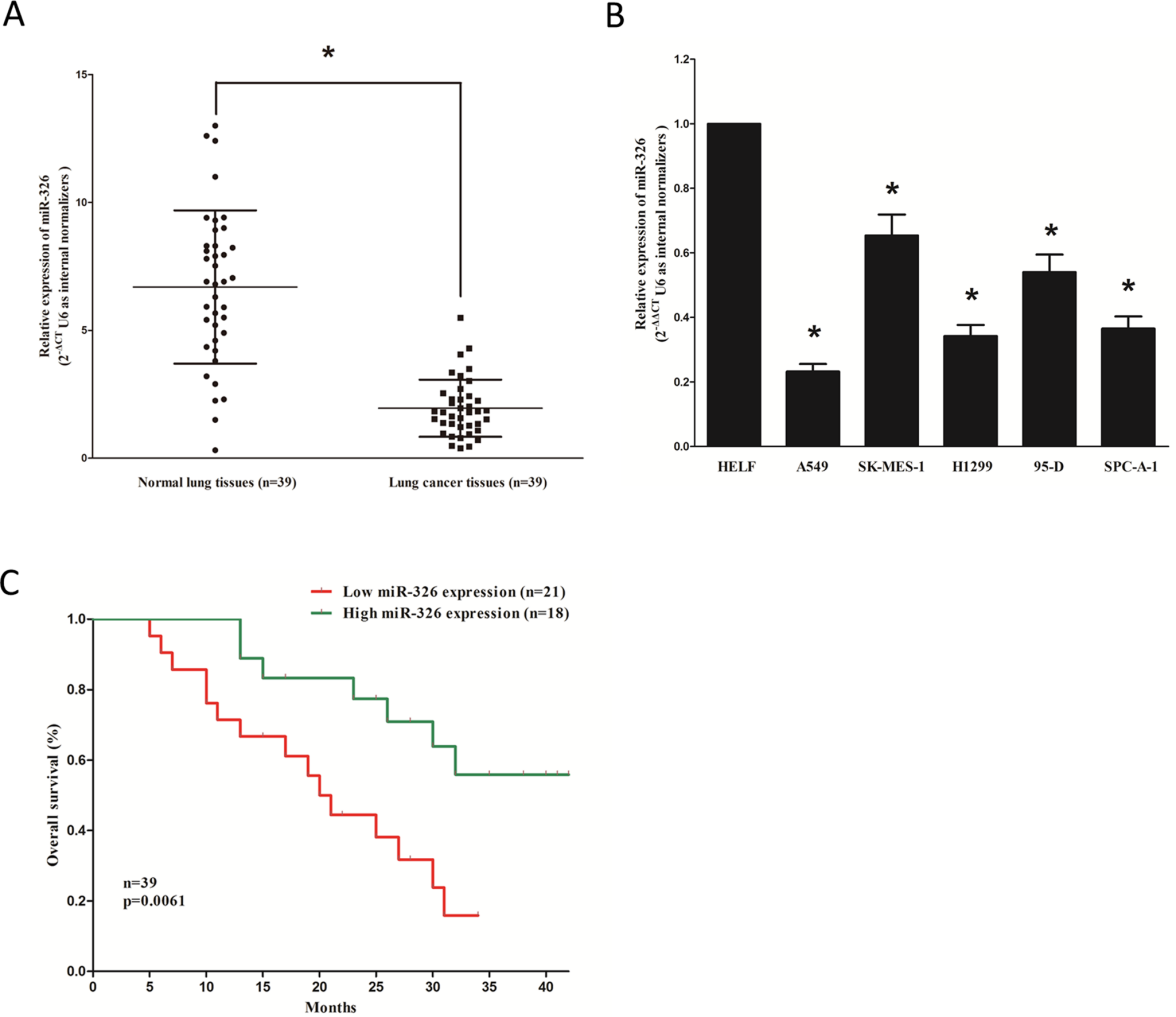
MiR-326 is down-regulated in primary human lung cancer and NSCLC cell lines, and benefits for prognosis (**A**) miR-326 is significantly decreased in primary human lung cancer tissues in comparison to adjacent-normal lung cancer tissues. *n* = 39 for each group. (**B**) The expression level of miR-326 in five NSCLC cell lines and normal HELF cells. Assays were performed in triplicate. (**C**) Kaplan-Meier survival analysis revealed that down-regulated miR-326 is associated with poor prognosis in patients with non-small cell lung cancer. Means ± SEM was shown. Statistical analysis was conducted using student *t*-test.

To determine whether miR-326 is down-regulated in lung cancer, we measured the mature miR-326 level in human primary lung tumors (NSCLC) and pair-matched adjacent lung normal tissues by qRT-PCR. We used U6 that is not deregulated in lung cancer for normalization. The results showed that miR-326 expression in the tumors was significantly (*P* < 0.05) reduced (mean = 29% of decrease) in 39 lung cancers relative to their matched controls among 39 samples analyzed (Figure 1A). Next, we examined miR-326 expression in NSCLC cell lines, and results demonstrated a lower expression of miR-326 in A549, H1299, 95D and SPC-A-1 cell lines, compared with that of in normal lung cells HELF (Figure 1B). Additionally, Kaplan–Meier survival analysis revealed that patients with low expression levels (≤ 29% of decrease, *n* = 18) of miR-326 had shorter overall survival, when compared with patients with high expression levels (> 29% of decrease, *n* = 21) of miR-326 (Figure 1C). These results demonstrated that down-regulation of miR-326 was associated with poor prognosis. Thus, it was concluded that the decreased expression of miR-326 might play an important role in lung cancer progression and development.

### Expression of cyclin D1 is up-regulated in primary human lung cancer and negatively expressed related to miR-326

**Figure 2:**
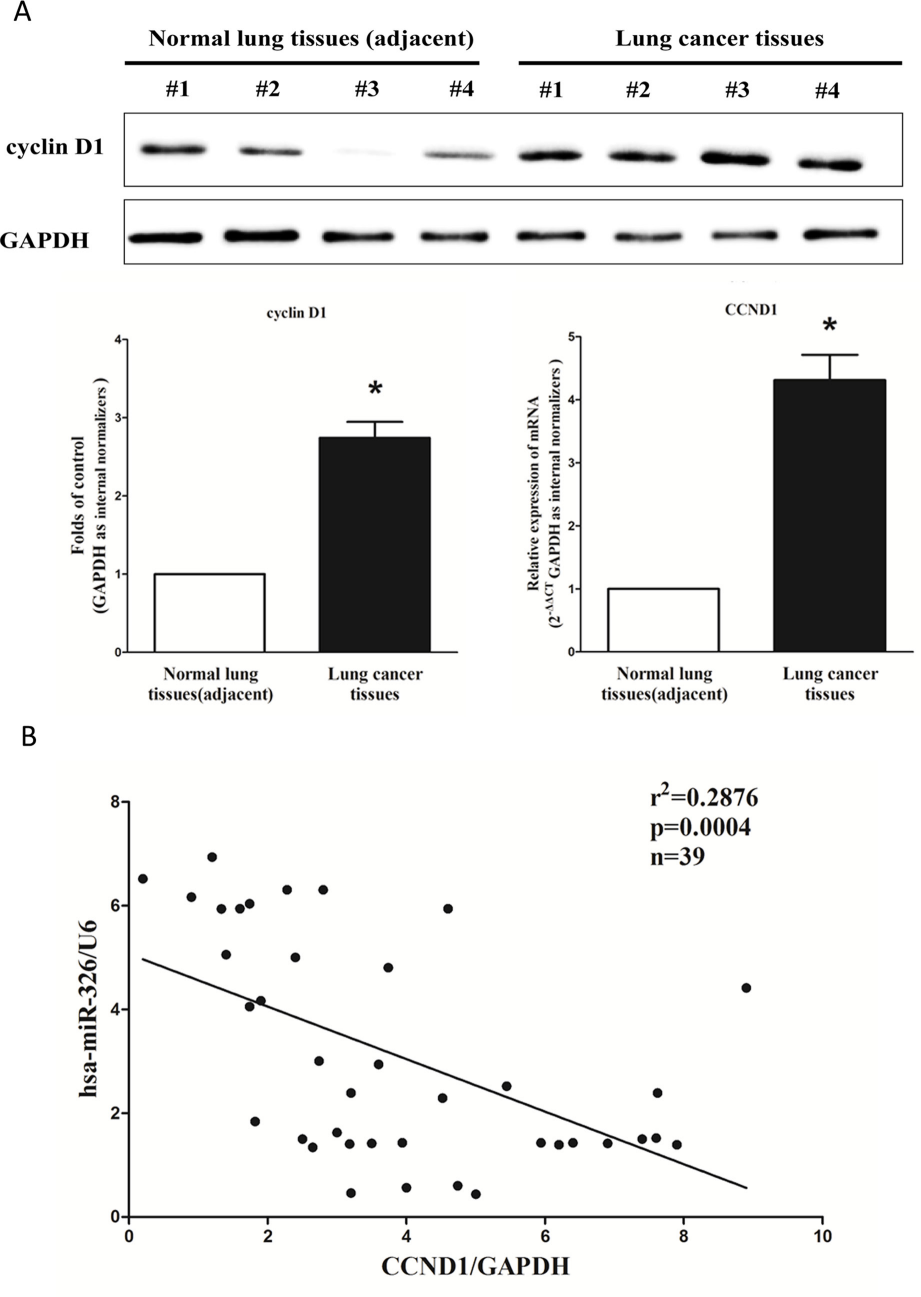
Expression of CCND1 is up-regulated in primary human lung cancer and negatively expressed related to miR-326 (**A**) Western-blot of cyclin D1 protein and qRT-PCR of CCND1 mRNA in lung cancer tissues and adjacent-normal lung cancers. *n* = 39 for each group. (**B**) Scatter plots showing the inverse association between miR-326 level and CCND1 mRNA expression. Means ± SEM was shown. Statistical analysis was conducted using student *t*-test.

cyclin D1 is important oncogene that shown strong power of oncogenicity, by promotion of cell growth, migration, invasion and epithelial mesenchymal transition (EMT), as well as inhibition of cell apoptosis in many tumors including lung cancer [35, 41, 42]. Thus, we next examined cyclin D1 expression in human primary lung tumors (NSCLC) and pair-matched adjacent lung tissues, and our western blot results demonstrated that the expression of cyclin D1 protein was increased in lung cancer tissues compared with normal lung tissues (Figure 2A). These results were confirmed by qRT-PCR of cyclin D1 mRNA expression (Figure 2A). Since cyclin D1 is the key role on regulation of cell cycle, aberrations of these three proteins might contribute to human lung cancer. Moreover, we evaluated the correlation between CCND1 mRNA and miR-326 expression in 39 lung cancer tissues. Expression of CCND1 mRNA and miR-326 exhibited a significant inverse correlation as calculated by Pearson correlation (*r*^2^ = 0.2876, *P =* 0.0004) (Figure 2B).

### Inhibition of miR-326 does not reverse the anticancer efficacy of silence of *CCND1* expression *in vitro*

**Figure 3:**
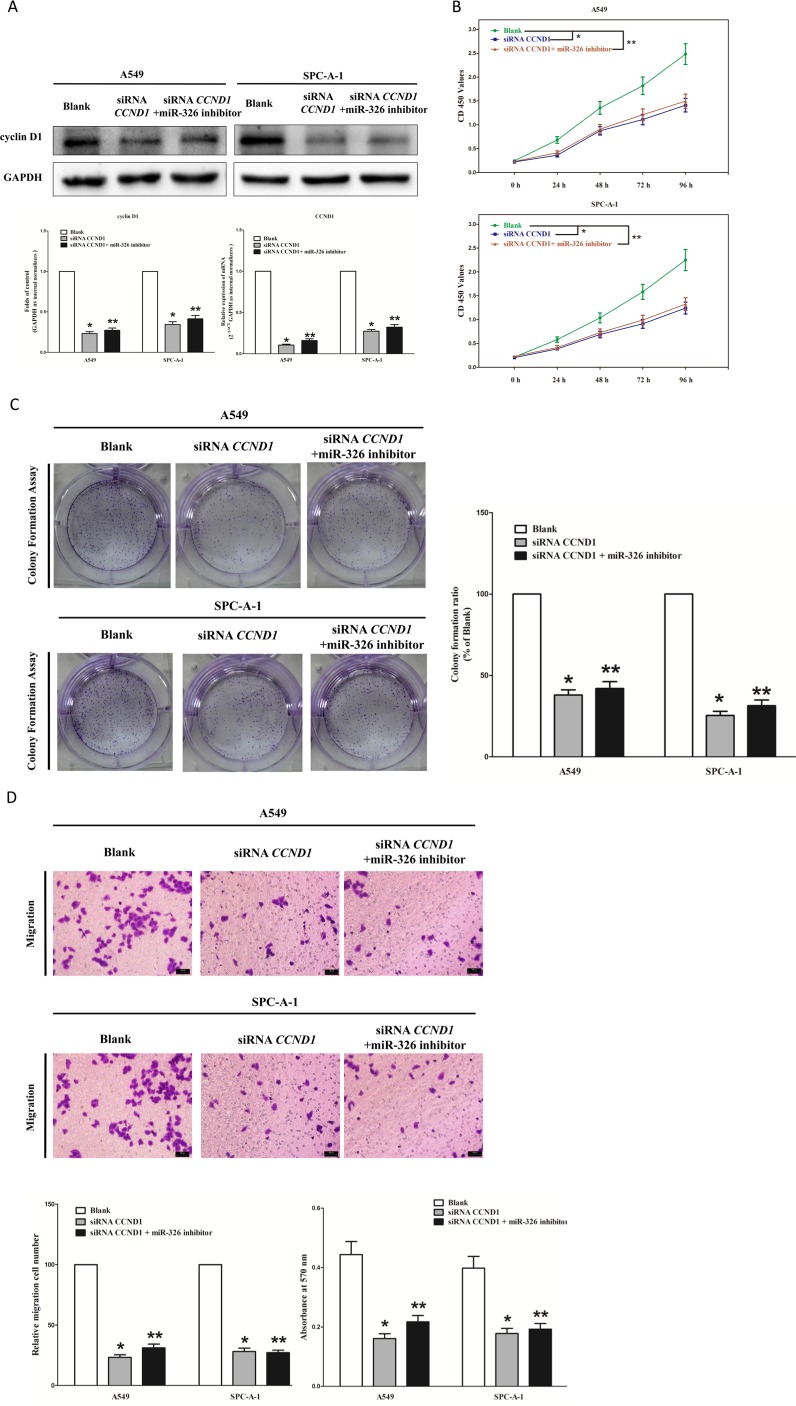

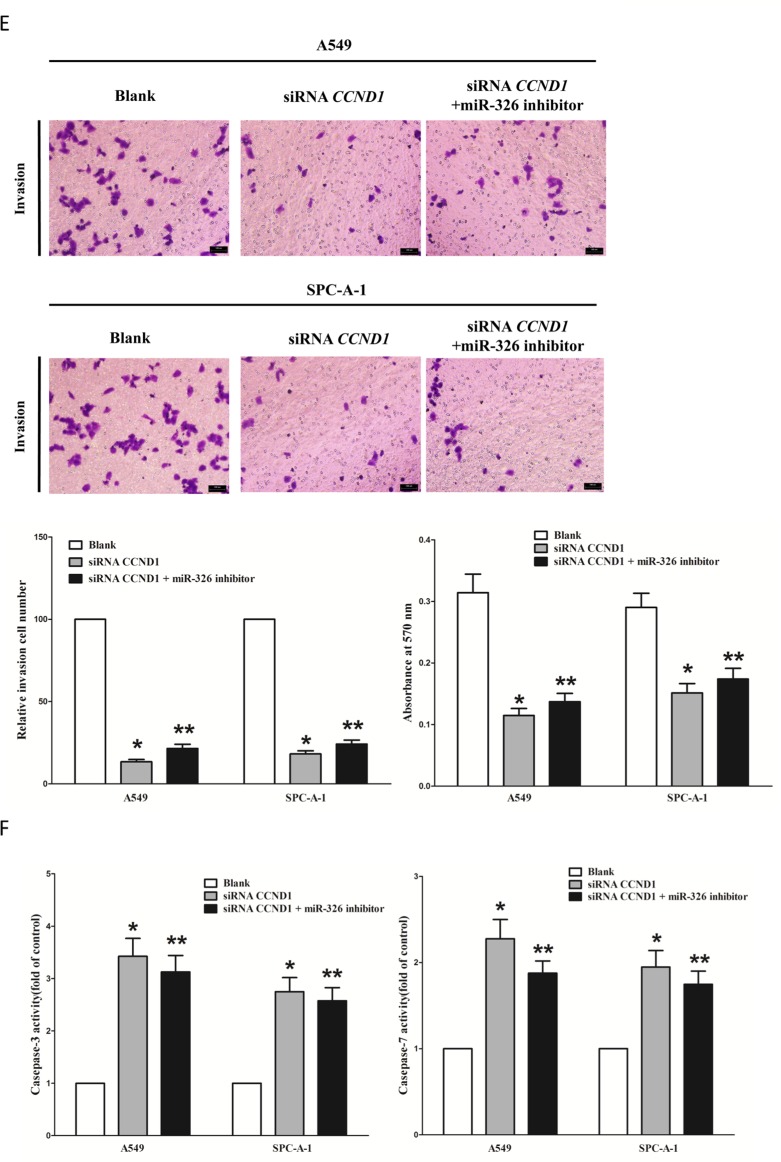
Inhibition of miR-326 does not reverse the anticancer efficacy of silence of CCND1 expression *in vitro* (**A**) Western-blot of cyclin D1 protein and qRT-PCR of CCND1 mRNA in siRNA *CCND1* treated and blank A549 and SPC-A-1 cells, and siRNA *CCND1 +* miR-326 inhibitor. (**B**) CCK8 assays of A549 and SPC-A-1 cells after transfected (un-transfected) with siRNA *CCND1* and siRNA *CCND1 +* miR-326 inhibitor. (**C**) Shown are representative photomicrographs of colony formation assay after transfected with (without) siRNA *CCND1* and siRNA *CCND1 +* miR-326 inhibitor for ten days. (**D**) Shown are representative photomicrographs of tanswell migration assay after transfected with (without) siRNA *CCND1* and siRNA *CCND1 +* miR-326 inhibitor. (**E**) Shown are representative photomicrographs of tanswell invasion assay after transfected with (without) siRNA *CCND1* and siRNA *CCND1 +* miR-326 inhibitor. (**F**) Quantitative representation of caspase-3 and caspase-7 activity in A549 and SPC-A-1 cells transfected with (without) siRNA *CCND1* and siRNA *CCND1 +* miR-326 inhibitor for forty eight hours. Assays were performed in triplicate. Means ± SEM was shown. Statistical analysis was conducted using ANOVA.

We next examined the potential tumorigenicity of *CCND1* in lung cancer. Silence of *CCND1* expression by siRNA significantly inhibited the expression of *CCND1* (Figure 3A). Moreover, loss of *CCND1* expression also contributed to inhibition of lung cancer cell (both A549 and SPC-A-1 cells) growth (Figure 3B and 3C) and metastasis (Figure 3D and 3E). In addition, inhibition of *CCND1* expression promoted apoptosis in lung cancer cell (both A549 and SPC-A-1 cells) (Figure 3F). These results further verified the powerful tumorigenicity of *CCND1* in lung cancer. Thus, we adopted *CCND1* for as targeted oncogenes. However, inhibition of miR-326 does not reverse the anticancer efficacy of silence of *CCND1* expression in lung cancer cell lines (both A549 and SPC-A-1 cells). These results indicate that the anticancer efficacy of miR-326 is partly attributed to it's inhibitory role on *CCND1*.

### MiR-326 targets human *CCND1*

**Figure 4:**
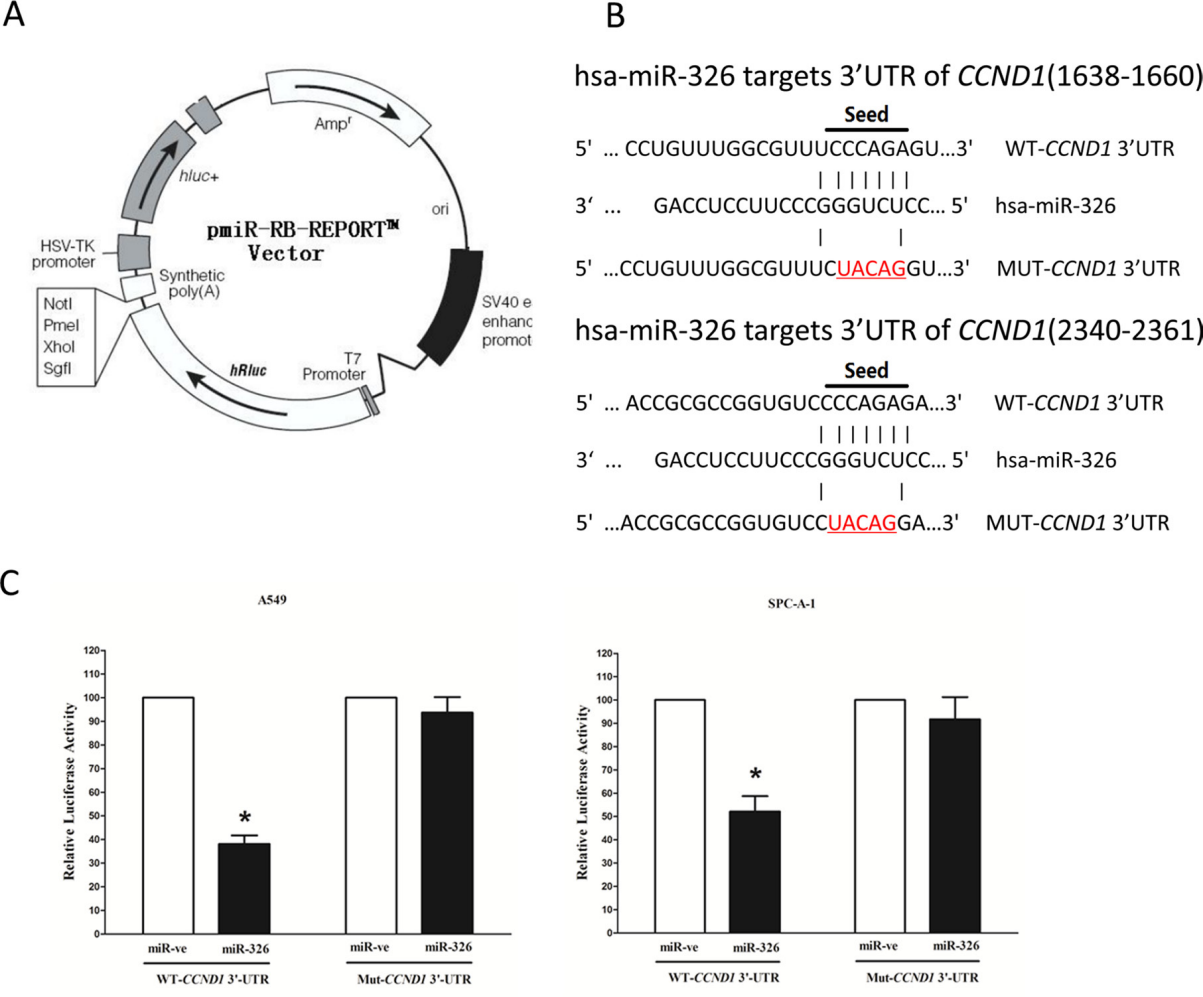
*CCND1* proto-oncogene is a target of miR-326 at specific 3′-UTR sites (**A**) pmiR-RB-REPORT^™^ dual-luciferase reporter vector. (**B**) The 3′-UTR of *CCND1* harbors two miR-326 cognate sites. (**C**) Relative luciferase activity of reporter plasmids carrying wild-type or mutant *CCND1* 3′-UTR in A549 and SPC-A-1 cells co-transfected with negative control (NC) or miR-326 mimic. Assays were performed in triplicate. Means ± SEM was shown. Statistical analysis was conducted using student *t*-test.

We then explored the underlying molecular mechanism of the antitumorigenic property of miR-326 in lung cancer cells. Since miRNAs primarily mediate their biological functions in animal cells by impeding the expression of target genes, we searched different data bases (TargetScan, microRNA.org and PicTar) for its potential targets that exhibited oncogenic properties. *CCND1*, which harbors two conserved miR-326 cognate sites, namely, 1668–1660 and 2340–2361 of *CCND1* 3′-UTR) (Figure 4B), is a predicted target of miR-326. To determine whether *CCND1* expression are indeed regulated by miR-326, the *CCND1* 3′-UTR were cloned into a luciferase reporter plasmid (Figure 4A), and the ability of miR-326 to inhibit expression of the adjacent hRluc coding region was quantified. For this purpose, the luciferase reporter plasmid pmiR-RB-REPORT^™^-*CCND*1-3′-UTR or a mutant reporter plasmids carrying point mutations in the putative miR-326 binding sites was co-transfected with miR-326 mimics or miR mimic NC, separately. The results show that miR-326 suppresses luciferase activity by approximately 62% in A549 cells and 49% in SPC-A-1 cells when the reporter plasmid carried the wild type *CCND1* 3′-UTR (Figure 4C), but no significant suppression was observed when the reporter plasmid carried a mutant *CCND1* 3′-UTR (i.e., pmiR-RB-REPORT^™^-mut-*CCND1*-3′-UTR). These results suggest that miR-326 binds directly to the predicted binding site in the *CCND1* 3′-UTR.

### MiR-326 suppresses tumor growth *in vivo*

**Figure 5:**
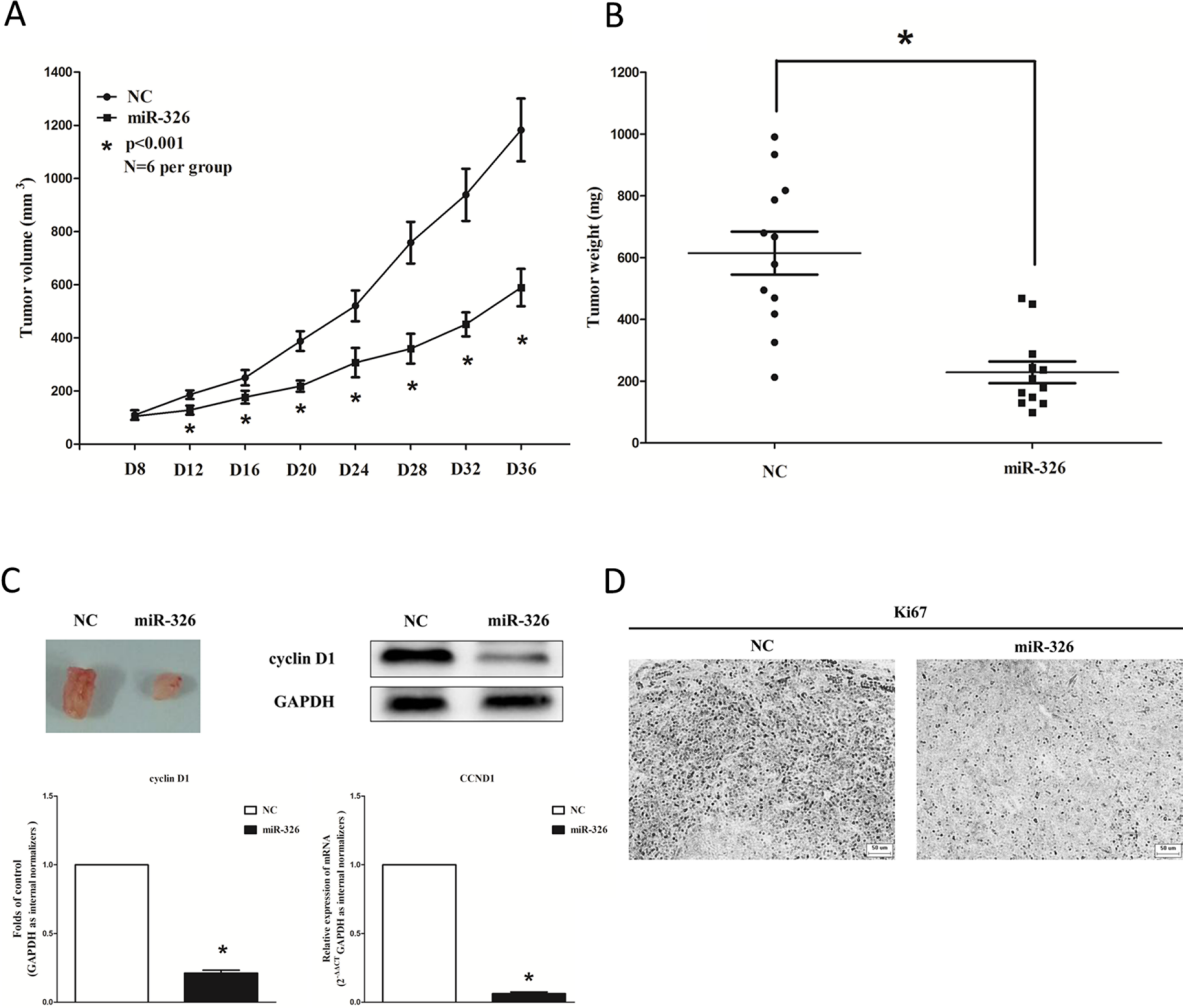
Ectopic expression of miR-326 suppresses tumor growth *in vivo* (**A**) Tumor volume in nude mice. (**B**) Tumor weight in nude mice. Each group contained six mice (*n* = 6); the data are presented as the mean ± SEM; *p < 0.001, compared with the NC group. (**C**) The expression of cyclin D1 protein and mRNA in nude mice. Assays were performed in triplicate. Means ± SEM are shown. Statistical analysis was conducted using student *t*-test. (**D**) Immunohistochemistry showed miR-326 decreased the proliferation index Ki67. Statistical analysis was conducted using student *t*-test.

To confirm the tumor suppressor role of miR-326 *in vivo*, we established a BALB/c nude mouse xenograft model using A549 cells. After 8 days, miR-326 agomir or miR agomir NC was directly injected into the implanted tumor every 4 days for seven times. The tumor volume was measured every 4 days until day 36. The tumor volume and weight of mice treated with miR-326 agomir were significantly reduced relative to that of treated with miR agomir NC (Figure 5A and 5B). This result indicates that miR-326 significantly inhibits the tumorigenicity of A549 cells in the nude mouse xenograft model. In addition, our results of western-blot and qRT-PCR demonstrated that the decreased expression of cyclin D1 in tumors developed from miR-326-agomir-treated nude mice relative to that of control tumors (Figure 5C). Moreover, immunohistochemical staining of resected tumor tissues found that tumors formed from miR-326-transfected A549 cells exhibited reduced positivity for Ki67 compared with those formed from control cells (Figure 5D). Thus, miR-326 reduces the growth of established non-small cell lung cancer xenografts.

### MiR-326 inhibits lung cancer cell proliferation and colony formation

**Figure 6:**
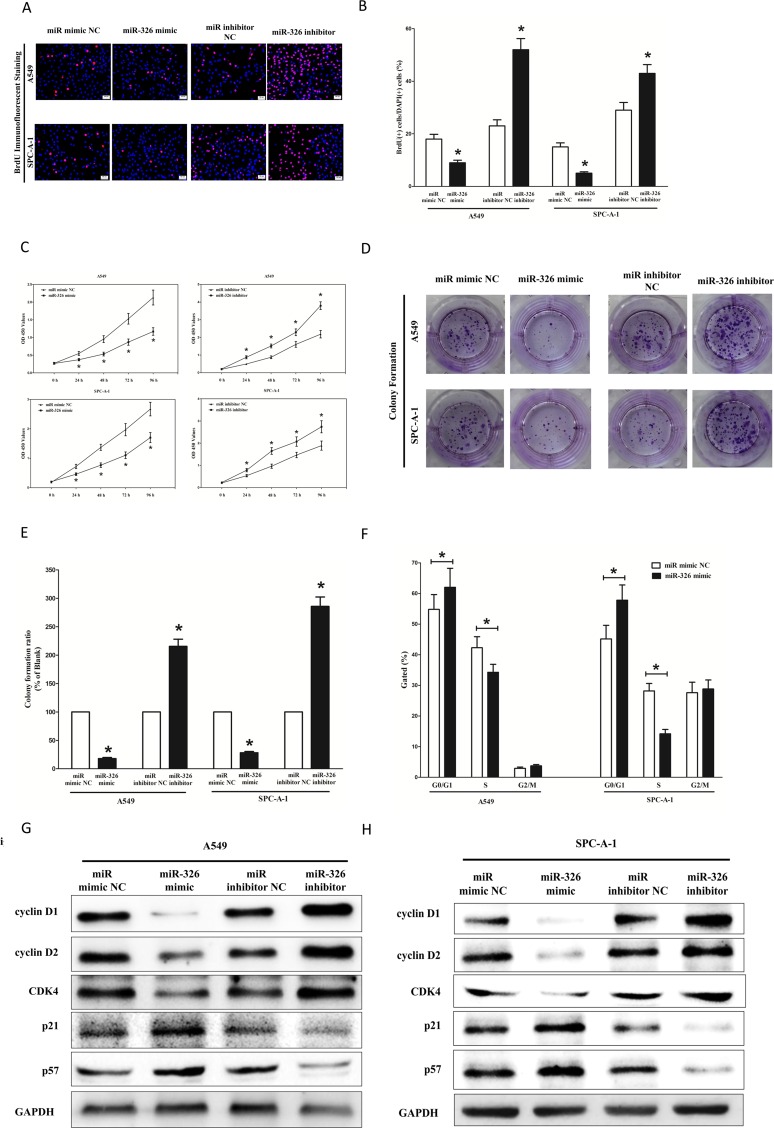
Ectopic expression of miR-326 inhibits proliferation and colony formation of A549 and SPC-A-1 cells (**A**) Shown are representative photomicrographs of BrdU staining after transfected A549 and SPC-A-1 cells with miR-326 mimic, miR-326 mimic NC, miR-326 inhibitor or miR-326 inhibitor NC for 24 h. Bar = 50 μm. (**B**) Statistical analysis of BrdU staining. (**C**) CCK8 assays of A549 and SPC-A-1 cells after transfected with miR-326 mimic, miR-326 mimic NC, miR-326 inhibitor, miR-326 inhibitor NC. (**D**) Shown are representative photomicrographs of colony formation assay after transfected with miR-326 mimic, miR-326 mimic NC, miR-326 inhibitor or miR-326 inhibitor NC for ten days. (**E**) Statistical analysis of colony formation assay. Assays were performed in triplicate. (**F**) Cell-cycle analysis was performed 48 h following the treatment A549 and SPC-A-1 cells with miR-326 mimic or miR-326 mimic NC. The DNA content was quantified by flow cytometric analysis. (**G–H**) Expression of cyclin D1, cyclin D1, CDK4, p21 and p57 protein in transfected A549 and SPC-A-1 cells. Assays were performed in triplicate. Means ± SEM was shown. Statistical analysis was conducted using student *t*-test.

To further investigate the anticancer role of miR-326 in lung cancer, we examined the role of miR-326 on NSCLC cell (A549 and SPC-A-1) proliferation. Our results of BrdU staining revealed that miR-326 inhibited A549 and SPC-A-1 cell DNA synthesis by approximately 45% (Figure 6A and 6B) and 62% (Figure 6A and 6B), compared with blank A549 and SPC-A-1 cells, respectively. However, miR-326 inhibitor treatment increased A549 and SPC-A-1 cell DNA synthesis by approximately 2.4 folds (Figure 6A and 6B) and 1.3 folds (Figure 6A and 6B) compared with blank A549 and SPC-A-1 cells, separately. To verify these results, we also did the CCK8 assay, and results demonstrated that miR-326 over-expression significantly attenuated A549 and SPC-A-1 cells vitality, while loss of miR-326 promoted cell proliferation (Figure 6C). In addition, we used colony formation assay to investigate the role of miR-326 on clonogenic survival, and results demonstrated miR-326 mimic treatment caused a decrease in the clonogenic survival of A549 cells compared with blank A549 cells (Figure 6D and 6E), while miR-326 inhibitor-treated A549 cells showed an significant increase in the clonogenic survival, when compared with blank A549 cells (Figure 6D and 6E). Furthermore, the growth inhibitory role of miR-326 on A549 and SPC-A-1 cells was accompanied by a corresponding increase in the proportion of cells in G1 and a decrease in the proportion of cells in the S phase (Figure 6F).

We next examined the influence of miR-326 on the expression of cyclin D1, a well-established human oncogene [41], which is over-expressed in lung cancer, breast cancer and pancreatic cancer [41–44], and over-expression of cyclin D1 is involved in malignant transformation in lung tissue [35]. Our results discovered that miR-326 significantly decreased the protein expression of cyclin D1, while loss of miR-326 remarkably increased the level of cyclin D1 in A549 and SPC-A-1 cells (Figure 6G). cyclin D2 is highly expressed and promotes tumorigenesisin numerous tumors [45, 46]. In our research, the protein expression of cyclin D2 was repressed by over-expression of miR-326 (Figure 6G). CDK4 amplification has been observed in several malignancies including glioma, breast cancer, and lung cancer [24], and an absence of CDK4 amplification in WD and DD liposarcomas is associated with lower rate of recurrence and favorable prognosis [47]. In our research, the protein expression of CDK4 was repressed by over-expression of miR-326 in A549 and SPC-A-1 cells (Figure 6G). p57 is a cyclin-dependent kinase inhibitor, and it is considered to be a candidate of tumor suppressor gene that has been implicated in cancers [48]. Our study revealed that the over-expression of miR-326 is a mechanism for the up-regulation of p57 level in NSCLC cell lines (A549 and SPC-A-1) (Figure 6G). The cell cycle inhibitor p21 has been shown to inhibit proliferation both *in vitro* and *in vivo* [49], and introduction of p21 expression constructs into normal [50] and tumor cell lines [51] results in cell cycle arrest in G1 [52]. Our study revealed that miR-326 up-regulated p21 level in NSCLC cell lines (A549 and SPC-A-1) (Figure 6G).

Collectively, these results clearly revealed that miR-326 markedly inhibited cell growth in lung cancer cells.

### MiR-326 inhibits lung cancer cell migration and invasion

Then, we examined the role of miR-326 on A549 and SPC-A-1 cells migration and invasion. Invasion and migration through the basement membrane are characteristics of metastatic cancer cells.

**Figure 7:**
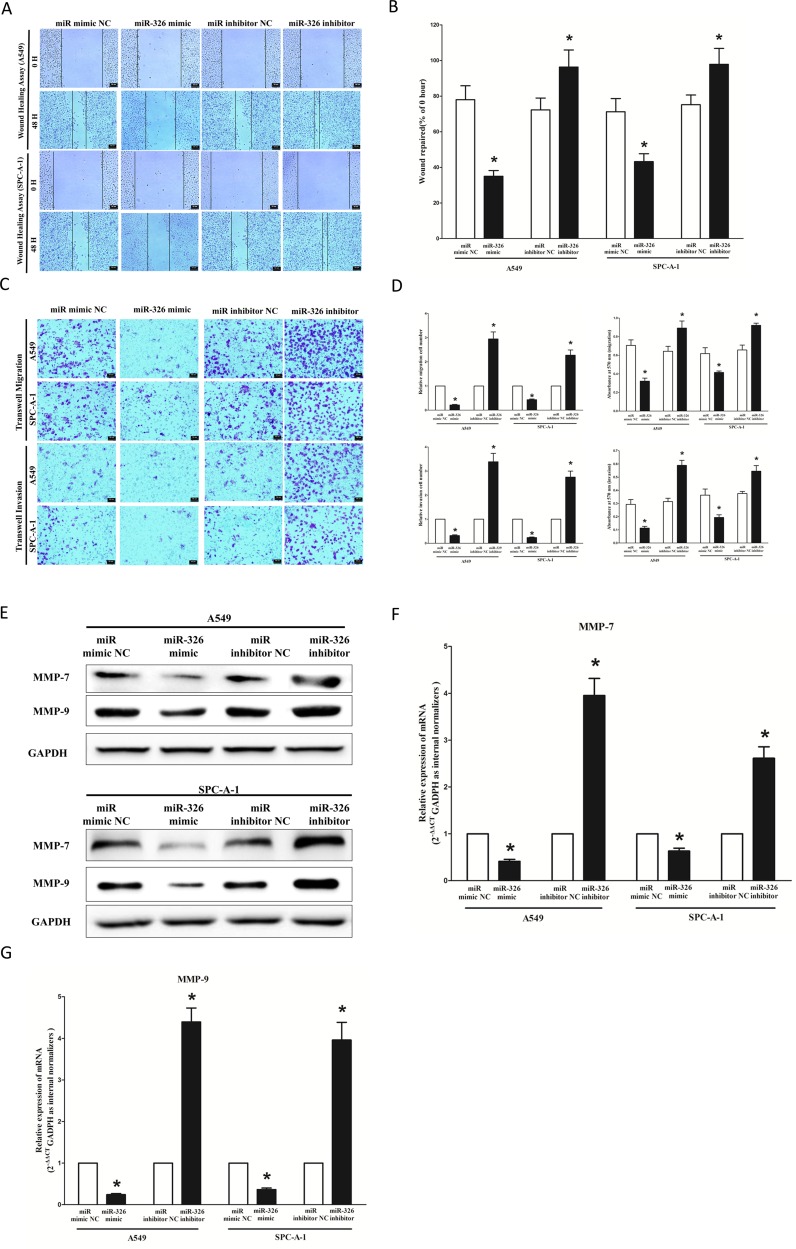
Ectopic expression of miR-326 in A549 and SPC-A-1 cells reduces cell migration and invasion motility (**A**) Shown are representative photomicrographs of “wound healing assay” in A549 and SPC-A-1 cells after transfected miRNAs for 0 hour and forty eight hours. Bar = 50 μm. (**B**) Statistical analysis of “wound healing assay”. (**C**) A549 and SPC-A-1 cells were loaded onto the top well of a transwell inserts for cell migration or invasion assay. After twenty four hours, cells that migrated to the bottom chamber containing serum-supplemented medium were stained with 0.1% crystal violet, visualized under a phase-contrast microscope, and photographed. Bar = 50 μm. (**D**) Total number of cells in five fields was counted manually. (**E**) Expression of MMP-7 and MMP-9 protein in A549 and SPC-A-1 cells after transfection. (**F–G**) Expression of MMP-7 and MMP-9 mRNA in A549 and SPC-A-1 cells after transfection. Assays were performed in triplicate. Means ± SEM was shown. Statistical analysis was conducted using student *t*-test.

We used two different approaches to assess the role of miR-326 on the ability of A549 and SPC-A-1 cells migration. In the first technique, we used a “scratch wound healing” assay. Motility of cells at different time points after generation of the wound was monitored under a microscope. Closure of the wound was complete within forty eight hours in control A549 and SPC-A-1 cells (Figure 7A and 7B). In contrast, miR-326-expressing cells migrated toward the wound at a much slower rate (Figure 7A and 7B). In the second approach, cells were seeded in serum-free medium on the top chamber of a two-chamber trans-well cell culture plate, and the cells migrated to the lower chamber containing complete medium after twenty four hours were photographed (Figure 7C) and counted. As expected, migration of miR-326-expressing clones was inhibited by 69% in A549 and 47% in SPC-A-1 cells, compared with the blank A549 and SPC-A-1 cells (Figure 7C and 7D), respectively. However, when treated with miR-326 inhibitor, migration in miR-326-expression defect A549 and SPC-A-1 cells were significantly increased by approximately 3 and 2.3 folds relative to blank A549 and SPC-A-1 cells (Figure 7C and 7D) respectively.

To investigate the role of miR-326 on A549 and SPC-A-1 cells invasion, we used a transwell invasion assay. As expected, invasion of miR-326-expressing clones was inhibited by 55% in A549 and 63% in SPC-A-1 cells, relative to the blank A549 and SPC-A-1 cells (Figure 7C and 7D), respectively. However, when treated with miR-326 inhibitor, invasion in miR-326-expression defect A549 and SPC-A-1 cells were significantly increased by approximately 3.4 and 2.7 folds relative to blank A549 and SPC-A-1 cells (Figure 7C and 7D), separately.

We also investigated the role of miR-326 on expression of MMP-7 and MMP-9, which all play a key role on tumor metastasis, and results indicated miR-326 inhibited the mRNA expression of MMP-7 and MMP-9 both in A549 and SPC-A-1 cells (Figure 7E and 7F). As expected, loss of miR-326 significantly increased the mRNA expression of MMP-7 and MMP-9 in both A549 and SPC-A-1 cells (Figure 7E and 7F).

These results, taken together, clearly demonstrated that miR-326 expression markedly reduces the migration and invasion motility of lung cancer cells.

### MiR-326 promotes lung cancer cell apoptosis

**Figure 8:**
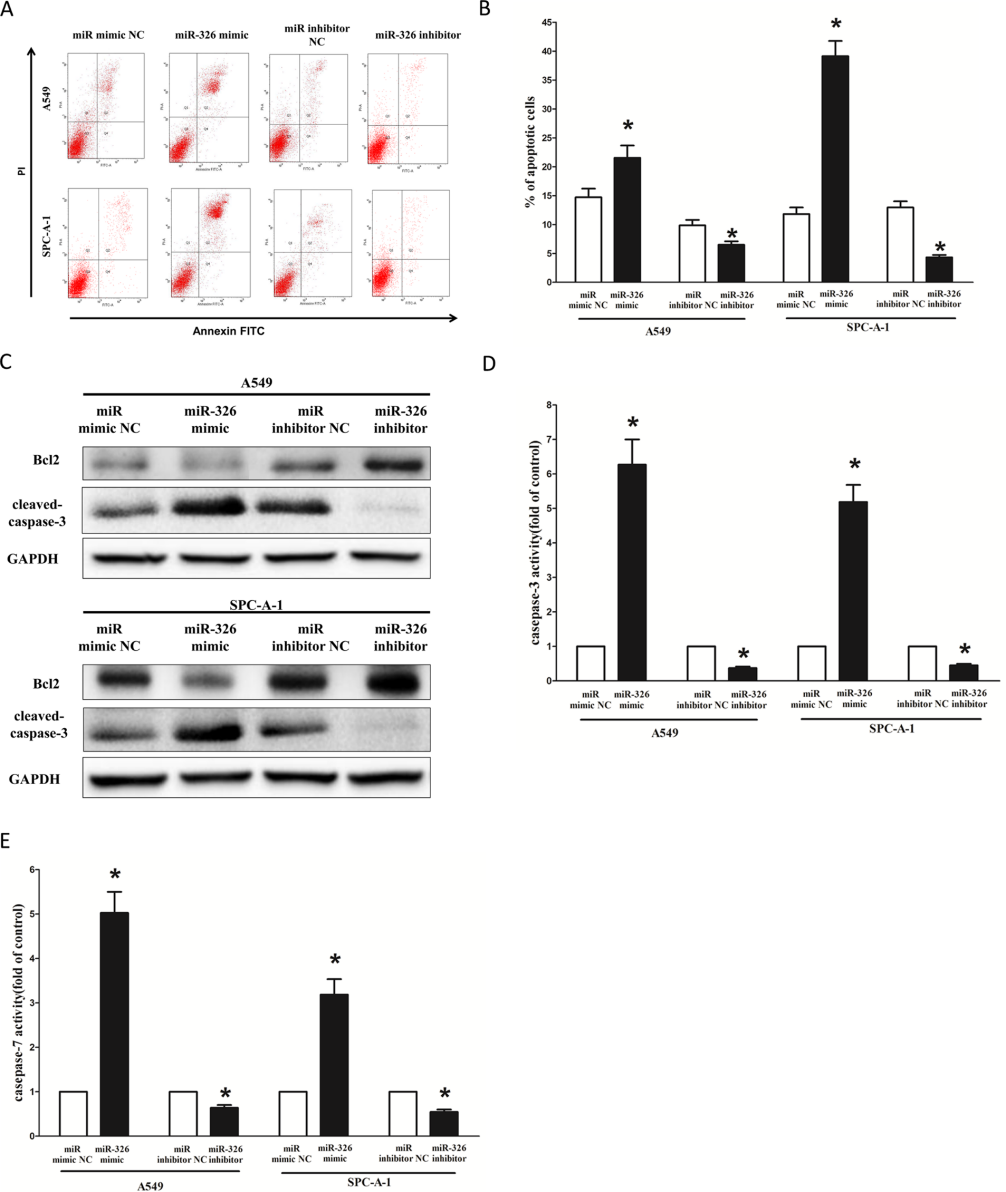
Ectopic expression of miR-326 promotes apoptosis in A549 and SPC-A-1 cells (**A**) Shown are representative photomicrographs of flow cytometric analysis. (**B**) Statistical analysis of flow cytometric analysis. (**C**) Western-blot of Bcl2 protein in A549 and SPC-A-1 cells after transfection. (**D–E**) Quantitative representation of caspase-3 and caspase-7 activity in A549 and SPC-A-1 cells transfected with related miRNAs for forty eight hours Assays were performed in triplicate Means ± SEM was shown. Statistical analysis was conducted using student *t*-test.

Next, we examined the role of miR-326 on A549 and SPC-A-1 cells apoptosis. Our results of flow cytometric analysis demonstrated that forced expression of miR-326 resulted in a ∼1.4-fold and ∼3.5-fold increase in apoptotic cell death of A549 and SPC-A-1 cells (Figure 8A and 8B), respectively. However, the percentage of apoptotic cells induced by miR-326 was decreased to the basal level when the cells were treated with the specific miR-326 inhibitor (Figure 8A and 8B). Moreover, miR-326 also inhibited the expression level of anti-apoptotic protein Bcl2 (Figure 8C), and increased the protein expression of cleaved-caspase-3 (Figure 8C) in A549 and SPC-A-1 cells. In addition, we also tested the caspase-3 and caspase-7 activity after treatment of A549 and SPC-A-1 cells with miR-326 mimic or miR-326 mimic NC, miR-326 inhibitor or miR-326 inhibitor NC, and results showed that miR-326 significantly increased the caspase-3 and caspase-7 activity in A549 and SPC-A-1 cell lysate, by approximately 6.2 and 5.5 folds increase (caspase-3 activity), 5.0 and 3.4 folds increase (caspase-7 activity), than that of in blank A549 and blank SPC-A-1 cells (Figure 8D and 8E), respectively. However, loss of miR-326 by transfecting with miR-326 inhibitor remarkably reduced the caspase-3 and caspase-7 activity in A549 and SPC-A-1 cell lysate, compared with that of in blank A549 and blank SPC-A-1 cells (Figure 8D and 8E), respectively. These results demonstrated that miR-326 indeed promoted apoptosis in A549 and SPC-A-1 cells.

## DISCUSSION

Our present study has revealed the following novel findings: (i) exogenously overexpressed miR-326 suppresses tumor regeneration in 6 lung cancer xenograft models and inhibits cell proliferation *in vitro* and *in vivo*; (ii) miR-326 overexpression inhibits lung cancer cells migration and invasion; (iii) inhibition of miR-326 in lung cancer cells results in high clonal, clonogenic, and tumorigenic properties; (iv) miR-326 overexpression promotes lung cancer cells apoptosis, and inhibition of miR-326 inhibits lung cancer cell apoptosis; (v) miR-326 targets *CCND1* in lung cancer cells and negatively expressed with *CCND1*.

Up to date, the molecular mechanisms underlying the development of lung cancers are still poorly understood. Therefore, a better understanding of the molecular mechanisms involved in tumor formation and development will be helpful to develop novel therapeutic targets and strategies for the treatment of human lung cancers. Although dysregulation of miRNAs was reported in various types of human cancers [53], aberrant expression and potential role of miRNAs in lung cancers were under studied. Down-regulation of miR-326 has been reported by miRNA profile studies on colorectal cancer [26], breast cancer [27], glioma [28–30], glioblastoma [31] and brain tumors [32]. Our data also indicated that miR-326 showed a reduced expression in lung cancer, suggesting the dysregulation of miR-326 is an early event of lung tumorigenesis.

We therefore characterized the putative tumor suppressive function of miR-326 in human lung cancer cell lines. Firstly, we examined the mechanism of miR-326 on lung cancer cell growth, and found that restoration of miR-326 in the lung cancer cell lines A549 and SPC-A-1 significantly inhibited cell proliferation as evidenced by BrdU, cell viability and colony formation assays. The growth-inhibition role of miR-326 may attribute to that miR-326 targets 3′-UTR of CCND1 mRNA, and inhibits the expression of CCND1 in lung cancer cells. In addition, miR-326 also inhibited cyclin D1, cyclin D2 and promoted p57 and p21 expression levels in lung cancer cells, which further contributed to the growth-delay efficacy of miR-326. In the present work, we showed that, in addition to inhibition of cell proliferation, the growth inhibitory effect of miR-326 was also related to induction of apoptosis. We observed that induction of miR-326 mediated apoptosis occurs by the modulation of extrinsic apoptosis pathway. Apoptosis induced by extrinsic pathways has been considered to be an important antitumor mechanism [54–56]. After transfection with miR-326, the expression of vital anti-apoptosis protein Bcl2 was down-regulated, and the activity of the downstream active apoptosis executor caspase-3 was up-regulated, leading to initiate a caspase cascade, and causing loss of DNA repair, cellular disassembly and finally apoptosis.

*In vitro* assays showed that restoration of miR-326 inhibited the cell migration and invasive capabilities. The reduced spreading effect and cell motility caused by miR-326 in lung cancer cells was revealed to be associated with the inhibition of the protein expression of cell migration and invasion molecules MMP-7 and MMP-9. MMP-7 and MMP-9 are members of the matrix metalloproteinases (MMPs) family, which are extracellular proteinases that regulate basic cellular processes including survival, migration and morpho-genesis and degradation extracellular matrix during the cancer metastatic process [57, 58]. MMP-7 is an established instigator of aggressive behavior in a number of cancer types including NSCLC. MMP-9 has been identified as a critical component for priming of the pre-metastatic niche. Thus, down-regulation of MMP-7 and MMP-9 expression by miR-326 contributed to dampened cell spreading and invasion ability.

Epigenetic silencing of miRNAs with tumor suppressor features is a common hallmark of human tumors. Having shown the crucial role of miR-326 in suppressing NSCLC development, we sought for the possible gene effectors participating in its function. Of note, a single miRNA can regulate a multitude of target genes concomitantly; for instance, it has been reported that miR-326 suppresses progression of colorectal cancer by down-regulating nin one binding protein [26]; and miR-326 could repress SMO oncogene in glioma [28]. Importantly, Cai *et al.* find that miR-326 promotes EMT-induced cells invasion in lung adenocarcinoma by down-regulation of Adam17 [59], but among the miRNAs predicted to target genes, we found that cyclin D1 acts as a critical effector of miR-326. We showed that miR-326 was able to significantly repress the luciferase activity of Luc-*CCND*1-3′ UTR by targeting the 3′ UTR of cyclin D1 mRNA. Therefore we focused on cyclin D1 for further analysis.

In our present study, we discovered that miR-326 was a potential prognostic marker for non-small cell lung cancer, and found miR-326 is dramatically down-regulated in human lung cancer tissues compared with normal lung tissues. Moreover, we also revealed up-regulation of miR-326 suppresses lung cancer cell proliferation, migration, invasion and colony formation, and promotes lung cancer cell apoptosis, through targeting cyclin D1. Our experimental data may provide a strategy for targeting the miR-326/cyclin D1 interaction in a novel therapeutic application to treat lung cancer patients.

## MATERIALS AND METHODS

### Tissue collection

Lung cancer tissues and adjacent normal lung tissues were obtained from patients who had undergone surgery at the People's Hospital of Wuhan University, between 2011 and 2015 and who were diagnosed with lung cancer based on histopathological evaluation. No local or systemic treatment had been conducted in these patients before the operation. All the tissue samples were collected, immediately snap frozen in liquid nitrogen, and stored at −80°C until RNA extraction. The study was approved by the Research Ethics Committee of Wuhan University (Wuhan, Hubei, PR China). Informed consent was obtained from all patients.

### Cell culture and transfection

The human NSCLC cell lines, namely, A549, SPC-A-1, H1299, SK-MES-1 and 95D cells were grown in RPMI 1640 (Gibco, USA), and HELF cells were grown in DMEM medium containing 10% heat-inactivated (56°C, 30 min) fetal calf serum, 2 mmol/L glutamine, penicillin (100 U/mL) and streptomycin (100 U/mL), which was maintained in an incubator at 37°C with 5% CO^2^ in a humidified atmosphere. Hsa-miRNA-326 mimic and mimic negative control, hsa-miRNA-326inhibitor and inhibitor negative control were purchased from GenePharma Co., Ltd. (Shanghai, China). For convenience, has-miRNA-326 mimic and mimic negative control, has-miRNA-326 inhibitor and inhibitor negative control were simply referred to as miR-326 mimic and miR mimic NC, miR-326 inhibitor and miR inhibitor NC, respectively. Complete medium without antibiotics was used to culture the cells at least twenty-four hours prior to transfection. The cells were washed with 1 × PBS (pH 7.4) and then transiently transfected with 50 nM miR-326 mimic or miR mimic NC, 100 nM miR-326 inhibitor or miR inhibitor NC, using Lipofectamine^™^ 2000 (Invitrogen, Carlsbad, CA, USA) according to the manufacturer's instructions.

### Western blot analysis

Forty-eight hours after transfection, total protein was extracted from the A549 and SPC-A-1 cells using RIPA cell lysis reagent containing proteinase and phosphatase inhibitors (Sangon Biotech, Shanghai, China) at 4°C for 30 min. Cell lysates were centrifuged at 12,000 × g for 20 min at 4°C, and the protein concentrations of the supernatant were determined using the BCA protein assay reagent kit (Thermo). The supernatants containing total protein were then mixed with a corresponding volume of 5 × SDS loading buffer and heated at 100°C for 10 min. Then, the supernatant lysates were run on 10% SDS-polyacrylamide gels (50 μg/lane), and proteins were transferred to poly (vinylidene fluoride) (PVDF) membranes (Hertfordshire, UK) by semidry electroblotting (1.5 mA/cm2). PVDF membranes were then incubated in blocking buffer [Tris-buffered saline (TBS) supplemented with 0.05% (vol/vol) Tween 20; TBST] containing 5% (wt/vol) skimmed milk powder for 120 min at room temperature followed by three 10 min washes in TBST. The PVDF membranes were then incubated with anti-cyclin D1 (1:1000 dilutions, Affinity), anti-cyclin D2 (1:1000 dilutions, Affinity), anti-CDK4 (1:1000 dilutions, Affinity), anti-p21 (1:1000 dilutions, Affinity), anti-p57 (1:1000 dilutions, Affinity), anti-MMP-7 (1:1000 dilutions, Affinity), anti-MMP-9 (1:1,000 dilutions, Affinity), anti-cleaved caspase 3 (1:1,000 dilutions, Affinity) and anti-GADPH (1:5,000 dilutions, Affinity) as internal normalizers in TBST containing 5% (wt/vol) skimmed milk powder (antibody buffer) overnight at 4°C on a three-dimensional rocking table. Then the membranes were washed three times for 10 min in TBST and then incubated with goat anti-rabbit IgG conjugated to horseradish peroxidase (1:12,000 dilutions) in antibody buffer for 120 min. Finally, membranes were washed three times for 10 min in TBST and exposed to ECL Advance reagent (GE Healthcare Biosciences, Buckinghamshire, UK) for 2 min as described in the manufacturer's protocol. Then membranes were exposed to Hyperfilm-ECL (GE Healthcare Bio-Sciences) for 2–5 min and visualized using a Fluor S Multimager and Quantity One 4.1 (Bio-Rad Laboratories, Hercules, CA). The molecular weights of the bands were calculated by a comparison with prestained molecular weight markers (molecular weight range: 6,500–250,000) that were run in parallel with the samples. Semiquantitative analysis of specific immunolabeled bands was performed using a Fluor S image analyzer and Quantity One 4.1.

### RNA isolation and quantitative reverse transcription poly-merase chain reaction (qRT-PCR)

Total RNA from the cultured cells was extracted using Trizol reagent (Invitrogen) according to the manufacturer's instructions. MiRNA levels were measured by qRT-PCR. For the qRT-PCR detection of mature miR-326 expression, we purchased the Bulge-Loop^™^ miRNA qRT-PCR Primer Set and the miRNA qRT-PCR Control Primer Set (both from RiboBio). RNA (2 μg) was converted into cDNA using the PrimeScript^™^ RT reagent kit with gDNA Eraser (Takara, Dalian, China) according to the manufacturer's instructions. qRT-PCR was performed using SYBR^®^ Premix Ex Taq^™^ II (Takara) in the ABI PRISM^®^ 7300 real-time PCR system (Applied Biosystems, Foster City, CA, USA). GADPH and U6 were used as endogenous controls. In addition, melting curves were used to evaluate non-specific amplification. The relative expression level was calculated using the 2^−ΔΔCt^ method. The primer sequences used in this study are as follows: the primers of miR-326 were purchased from RiboBio (RiboBio Co., Ltd, Guangzhou, China); human CCND1: sense: 5′-CTCCTCTCCGGAGCATTTTGATA-3′, antisense: 5′-T TAAAGACAGTTTTTGGGTAATCT-3′; human MMP-7: sense: 5′-GAGTGCCAGATGTTGCAGAA-3′, antisense: 5′-AAATGCAGGGGGATCTCTTT-3′; human MMP-9: sense: 5′-CTGCAGTGCCCTGAGGACTA-3′, antisense: 5′-ACTCCTCCCTTTCCTCCAGA-3′; The formula and its derivations were obtained from the ABI Prism 7300 sequence detection system user guide. Statistical analysis was performed on the fold change.

### Colony formation assay

Cells were transfected with miR-326 mimic or miR mimic NC, miR-326 inhibitor or miR inhibitor NC, as described above. Twenty-four hours later, transfected cells were trypsinized, counted and replated at a density of 500 cells/6 cm dish. Ten days later, colonies resulting from the surviving cells were fixed with 3.7% methanol, stained with 0.1% crystal violet and counted. Colonies containing at least 50 cells were scored. Each assay was performed in triplicates.

### Luciferase reporter assays

The 3′-untranslated region (UTR) of human *CCND1* was amplified from human genomic DNA and individually inserted into the pmiR-RB-REPORT^™^ (Ribobio, Guangzhou, China) using the XhoI and NotI sites. Similarly, the fragment of *CCND1* 3′-UTR mutant was inserted into the pmiR-RB-REPORT^™^ control vector at the same sites. For reporter assays, A549 cells were co-transfected with wild-type (mutant) reporter plasmid and miR-326 mimics (miR mimic NC) using Lipofectamine 2000 (Invitrogen). Firefly and Renilla luciferase activities were measured in cell lysates using the Dual-Luciferase Reporter Assay system. Luciferase activity was measured forty-eight hours post-transfection using dual-glo luciferase reporter system according to the manufacturer's instructions (Promega, Madison, WI, USA). Firefly luciferase units were normalized against Renilla luciferase units to control for transfection efficiency.

### Transwell migration/invasion assay

A549 and SPC-A-1 cells were grown in RPMI 1640 containing 10% fetal bovine serum to ∼60% confluence and transfected with 50 nM miR-326 mimic or a negative control, 100 nM miR-326 inhibitor or a negative control. After twenty-four hours, the cells were harvested by trypsinization and washed once with Hanks' balanced salt solution (Invitrogen). To measure cell migration, 8-mm pore size culture inserts (Transwell; Costar, High Wycombe, UK) were placed into the wells of 24-well culture plates, separating the upper and the lower chambers. In the lower chamber, 500 μL of RPMI 1640 containing 10% FBS was added. Then, serum-free medium containing 5 × 10^4^ cells were added to the upper chamber for migration assays, whereas 1 × 10^5^ cells were used for matrigel invasion assays. After twenty-four hours of incubation at 37°C with 5% CO^2^, the number of cells that had migrated through the pores was quantified by counting 10 independent visual fields under the microscope (Olympus) using a × 20 magnifications, and cell morphology was observed by staining with 0.1% crystal violet. Filters were washed thoroughly with 1 × PBS and dissolved in 500 μL of 33% acetic acid, and absorbance was measured at 570 nm. Absorbance of cells incubated in the serum-free medium in the bottom chamber was used as negative control. Each experiment was performed at least three times.

### BrdU immunofluorescence assay

A549 and SPC-A-1 cells were seeded on sterile cover glasses placed in the 6-well plates. After transfection with miR-326 mimic, miR mimic NC, miR-326 inhibitor, miR inhibitor NC for forty eight hours, the BrdU (5-bromo-2-deoxyuridine; Sigma) stock solution at 10 mg/mL in saline was diluted 1000 × in the culture medium and incubated for 60 min. After washing with 1 × PBS, cells were then fixed for 20 min in 4% paraformaldehyde (PFA) and permeabilized with 0.3% Triton X-100 for 10 min. After blocking with 10% goat serum in 1 × PBS for 1 h, cells were incubated with a primary rabbit antibody against BrdU (1:200, Abcam) over night at 4°C, and then incubated with the secondary antibody coupled to a fluorescent marker, Cy3, at room temperature for 2 h. After DAPI staining and 1 × PBS washing, the cover slips were mounted on to glass slides with anti-fade solution and visualized using a fluorescence microscope (Olympus 600 auto-biochemical analyzer, Tokyo, Japan) with Image-Pro Plus software for image analysis, and 10 microscopic fields were taken for calculating BrdU.

### CCK8 assay

Cell growth was measured using the cell proliferation reagent WST-8 (Roche Biochemicals, Mannheim, Germany). After plating cells in 96-well microtiter plates (Corning Costar, Corning, NY) at 1.0 × 10^3^/well, 10 μL of CCK8 was added to each well at the time of harvest, according to the manufacturer's instructions. One hour after adding CCK8, cellular viability was determined by measuring the absorbance of the converted dye at 450 nm.

### Transfection of siRNA

cyclin D1 siRNA was purchased from Santa Cruz (sc-29286). For transfection, the cells were plated on an antibiotic-free growth medium at 30–40% confluence approximately 24 h before transfection. RNA oligonucleotides were transfected using Lipofectamine^™^ 2000 (Invitrogen, USA) according to the manufacturer's protocol.

### Tumor formation in BALB/c nude mice

BALB/c athymic nude mice (male, 4–6-weeks old and 16–20 g) were purchased from Hubei Research Center of Laboratory Animal (Wuhan, China). All animal experiments were carried out in accordance with the Guide for the Care and Use of Laboratory Animals of Wuhan University. To establish lung cancer xenograft model, 5 × 10^5^ A549 cells were suspended in 100 μL phosphate-buffered saline and inoculated subcutaneously into the flanks of nude mice. After 8 days, the transplanted nude mice were randomly divided into two groups (*n* = 6 each). miR-326 agomir (miR-326) or miR agomir NC (NC) (RiboBio Co., Ltd, Guangzhou, China) was directly injected into the implanted tumor at the dose of 1 nmol (in 20 μL phosphate-buffered saline) per mouse every 4 days for seven times. The tumor size was monitored by measuring the length (L) and width (W) with calipers every 4 day, and the volumes were calculated using the formula: (L × W^2^)**/**2. Mice were killed by cervical dislocation in day 28, and the tumors were excised and snap-frozen for protein and RNA extraction.

### Immunohistochemistry

Immunohistochemistry of the tumor tissues was performed as described previously [36–40]. 3-μm tumor sections were incubated with commercial rabbit polyclonal antibodies against Ki67 (Affinity) at 1/100 dilution overnight at 4°C. Then, the sections were conjugated with horseradish peroxidase (HRP) antibody (1:500 dilution; Santa Cruz Biotechnology, Santa Cruz, CA) at room temperature for 2 h, then covered by DAB (Vector Laboratories, Burlingame, CA), and slides were mounted with Vectashield mounting medium (Vector Laboratories). Subsequently, all fields were observed under light microscopy (Olympus 600 auto-biochemical analyzer, Tokyo, Japan). Control experiments without primary antibody demonstrated that the signals observed were specific.

### Flow cytometry

A549 cells transfected with miR-326 mimic or negative control were trypsinized and resuspended in 1 × binding buffer at 1 × 10^6^ cells/mL. 100 μL of this cell suspension was incubated with 5 μL of FITC-Annexin V and 5 μL propridium iodide (PI) for 15 minutes in the dark. The reaction was terminated with the addition of 400 μL 1 × binding buffer and analyzed with (FACSCalibur using the CellQuest software (Becton Dickinson). FITC-Annexin V-positive and PI-negative cells were considered as apoptotic and the experiments were carried out in triplicates.

### Wound healing assay *in vitro*

The A549 and SPC-A-1 cells were seeded in 6-well plates and incubated for twenty-four hours. Then a linear wound was tehncreated by dragging a 100-μL pipette tip through the monolayer prior to transfection. Cellular debris was removed by gentle washes with culture medium, following which transfection was performed immediately, and the cells were allowed to migrate for a further forty-eight hours. The healing process was dynamically photographed after the wound was introduced using a microscope (Olympus 600 auto-biochemical analyzer, Tokyo, Japan). Migration distance was measured from images (5 fields) taken at each indicated time point. The gap size was analyzed using Image-Pro Plus 6.0 software. The residual gap between the migrating cells from the opposing wound edge was expressed as a percentage of the initial gap size.

### Caspase-3/7 activity assay

The activity of caspase-3/7 was determined using the caspase-3/7 activity kit (Beyotime Institute of Biotechnology, Haimen, China). To evaluate the activity of caspase-3/7, cell lysates were prepared after their respective treatment with various designated treatments. Assays were performed on 96-well microtitre plates by incubating 10 μL protein of cell lysate per sample in 80 μL reaction buffer (1% NP-40, 20 mM Tris-HCl (pH 7.5), 137 mM Nad and 10% glycerol) containing 10 μL caspase-3 substrate (Ac-DEVD-pNA) (2 mM). Lysates were incubated at 37°C for 4 h. Samples were measured with an ELISA reader at an absorbance of 405 nm. The detail analysis procedure was described in the manufacturer's protocol.

### Statistical analysis

All experiments were repeated 3 times independently. The results are presented as the means ± standard error mean (SEM). Two independent sample *t*-test or One-Way Analysis of Variance (ANOVA) was performed using SPSS 19.0 software in order to detect significant differences in measured variables among groups. A value of *P* < 0.05 was considered to indicate a statistically significant difference.

## SUPPLEMENTARY MATERIAL FIGURE



## The abbreviations used are: used

miR: microRNA
miR-326: has-microRNA-326
NSCLC: non-small cell lung cancer
3′-UTR: 3′-untranslated region
ERα: estrogen receptor alpha
BCL2: B-cell lymphoma-2
