# Supplementary material for: Hsa-miR-326 targets CCND1 and inhibits non-small cell lung cancer development
Source: Oncotarget. 2016 Jan 29;7(7):8341–59. doi: 10.18632/oncotarget.7071 (PMC4884997; doi:10.18632/oncotarget.7071)
Supplement: Supplementary file 1 [file oncotarget-07-8341-s001.pdf]

Hsa-miR-326 targets *CCND1* and inhibits non-small cell lung cancer development

Supplementary Materials

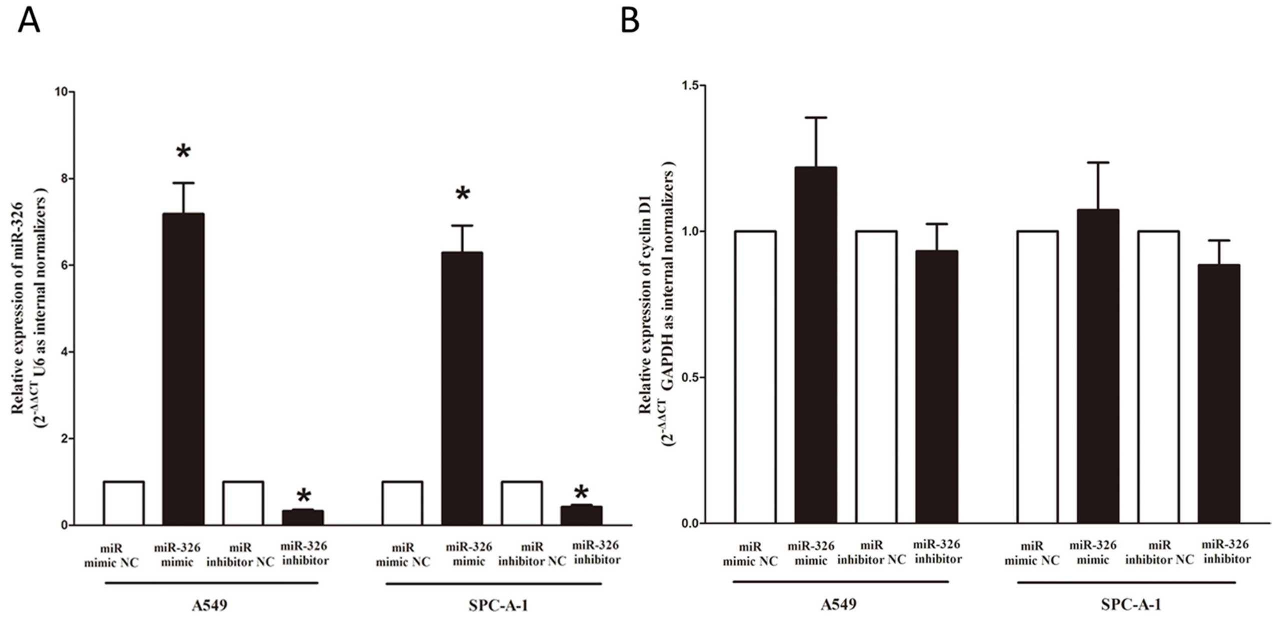

Supplementary Figure S1: Ectopic expression of miR-326 doesn't vary the mRNA levels of cyclin D1 in A549 and SPC-A-1 cells. (**A**) miR-326 mimic treatment increases the expression of miR-326, while miR-326 inhibitor treatment decreases it. (**B**) Ectopic expression of miR-326 doesn't vary the mRNA levels of cyclin D1 in A549 and SPC-A-1 cells. Assays were performed in triplicate Means ± SEM was shown. Statistical analysis was conducted using student *t*-test.
